# Supplementary figures and images for: Hantaan virus-derived peptides that stabilize HLA-E could abrogate inhibition of CD56dimNKG2A+ NK cells
Source: PLoS Pathog. 2025 Jul 18;21(7):e1012717. doi: 10.1371/journal.ppat.1012717 (PMC12303380; doi:10.1371/journal.ppat.1012717)

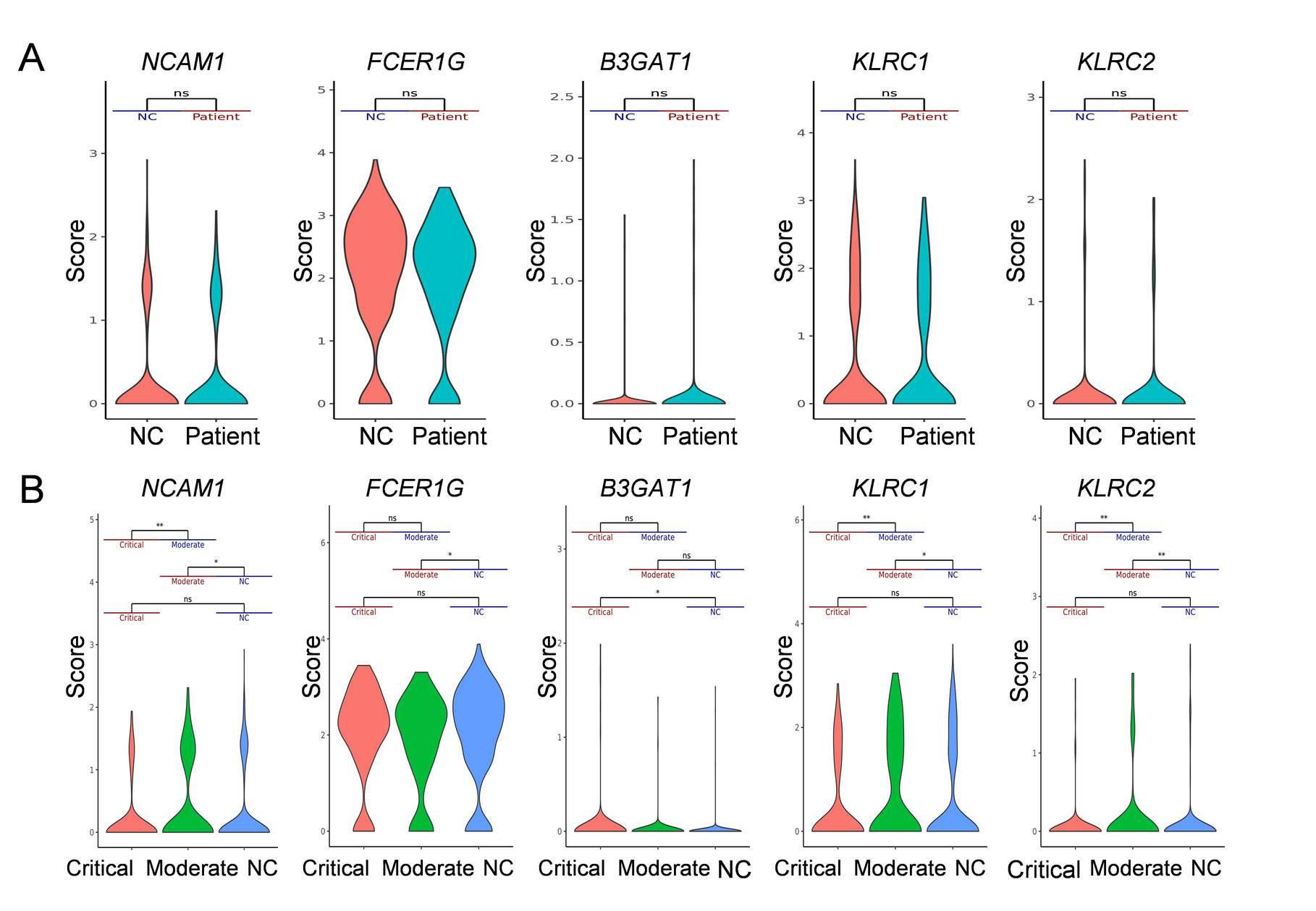

Supplement: S1 Fig — The violin graph showing the comparison of the above genes expression in total NK cells (A) between uninfected controls and HFRS patients; and (B) in HFRS patients with different disease severities and uninfected controls. (TIF) [file ppat.1012717.s001.tif]

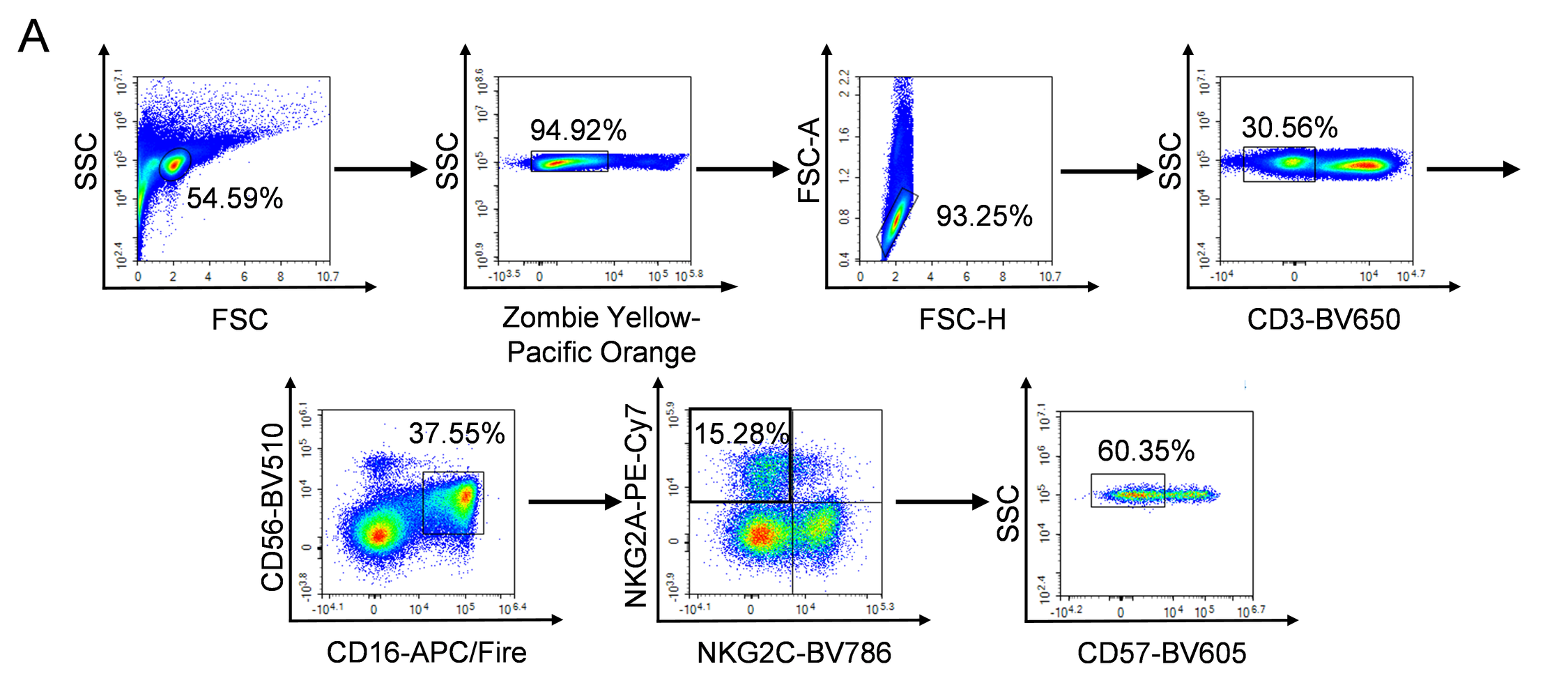

Supplement: S2 Fig — Based on the expression of CD3, CD56, CD16, NKG2A, NKG2C, CD57, NK cells were gated as CD3-CD56dimCD16+NKG2A+NKG2C-CD57- cells (Cells with the above phenotype were referred to as CD56dimNKG2A+ NK cells, which were the target subgroup of the research). (TIF) [file ppat.1012717.s002.tif]

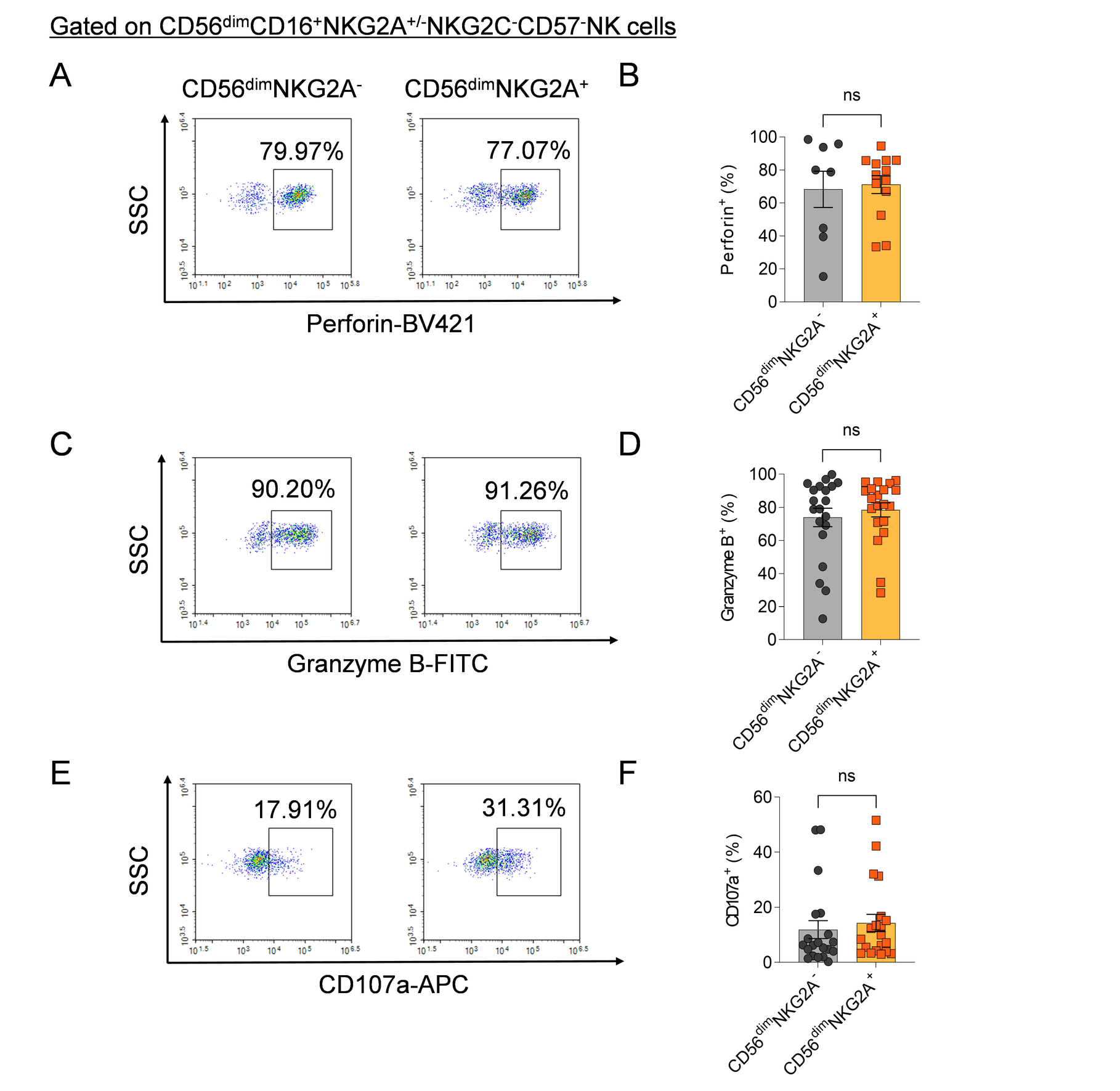

Supplement: S3 Fig — Representative flow cytometric plots and the comparison of the frequencies of perforin (A and B), granzyme B (C and D) and the expression of CD107a (E and F) on CD56dimCD16+NKG2A+/-NKG2C-CD57- NK cells. (TIF) [file ppat.1012717.s003.tif]
