# Supplementary material for: Hantaan virus-derived peptides that stabilize HLA-E could abrogate inhibition of CD56dimNKG2A+ NK cells
Source: PLoS Pathog. 2025 Jul 18;21(7):e1012717. doi: 10.1371/journal.ppat.1012717 (PMC12303380; doi:10.1371/journal.ppat.1012717)
Supplement: Attachment — Submitted filename: Response to Reviewers.docx [file ppat.1012717.s007.docx]

**Replies to Reviewers and Editors**
First of all, we thank both reviewers and editors for their positive and constructive comments and suggestions. Below, the original comments are in black, and our responses are in blue. All the pages and lines in our responses are indicated in the file of “Revised Article with Changes Highlighted”.

**Comments from the Editors:**

As the comments from the reviewers, the study offers valuable insights into how HTNV-derived peptides interact with NK cells and impact the function, which could inform future therapeutic developments. However, some concerns are also raised by the reviewers. The following comments and suggestions from the reviewers may benefit the revision of the work for the authors.

1. The authors need to make clearer analysis and explanation for the following results, such as the unusual detection of MHC class II molecular HLA-DR expression, high CD69 expression in NK cells of HFRS patients at convalescence stage.

**Response:** Thank you for pointing out this important issue. We accept the valuable suggestion, and we have tried our best to address them one by one in detail as follow. First, numerous studies assessed the late activation status of NK cells by analyzing expression of human leukocyte antigen-DR (HLA-DR) in infectious diseases (refs 1-3). Second, a previous publication reported that activated HLA-DR^+^ NK cells were detected in both healthy donors and SARS-CoV-2-infected individuals at acute or convalescent stages. The highly activated NK cells exhibited a dynamic pattern, reaching their peak within the first 3 to 4 weeks after symptom onset and subsequently declining (ref 4). Therefore, HLA-DR could be regarded as the late activation markers for NK cells from the peripheral blood of hemorrhagic fever with renal syndrome (HFRS) patients.

CD69 is an early activation marker expressed on activated NK cells (ref 5). For example, as Dengue viral (DENV) infection progresses, the activated CD69^+^CD56^dim^ NK cells remain detectable in patients with severe dengue even during the recovery phase. The CD69^+^CD56^dim^ NK cells are significantly elevated compared to healthy controls, although their levels are lower than those observed in DENV-infected patients during the acute phase (ref 6). Therefore, in our study, we detected the expression of CD69 on CD56^dim^NKG2A^+^NK cells in PBMCs from 19 patients during the acute phase and 7 patients during the convalescent phase.

In the revised manuscript, we have taken this weakness into consideration and made clearer analysis of the proportion of CD69^+^CD56^dim^NKG2A^+^ NK cells in PBMCs from HFRS patients with different disease stages and uninfected controls. The data mentioned above have been added into the Results section in revised manuscript (Page 10, Line 208 to Line 218) as below and presented as a new figure (Figs 4A-D). Moreover, a new discussion part has been added to better explain the unusual detection of HLA-DR expression, high CD69 expression in NK cells of HFRS patients at convalescence stage in Page27, Line 516 to Page28, Line 523 as follow.

Page 10, Line 208 to Line 218:

“Next, the activation status of CD56^dim^NKG2A^+^NK cells in HFRS patients were detected. Notably, the frequencies of HLA-DR^+^CD56^dim^NKG2A^+^NK cells in both mild/moderate and severe/critical HFRS patients were significantly higher than that in uninfected controls (*p*<0.01) (Figs 4A and B). The frequencies of CD69^+^CD56^dim^NKG2A^+^NK cells seem to be higher in both mild/moderate and severe/critical HFRS patients than that in uninfected controls, but showed no statistical difference among them. When comparing patients at different stages of the disease, the frequency of CD69^+^CD56^dim^NKG2A^+^NK cells was especially higher at convalescent stage than that at acute stage (*p*<0.01) (Figs 4C and D). The frequencies of both HLA-DR^+^CD56^dim^NKG2A^+^NK cells and CD69^+^CD56^dim^NKG2A^+^NK cells were higher at acute stage or convalescent stage of HFRS patients than that in uninfected controls (*p*<0.01 and *p*<0.001, respectively).”

Page27, Line 516 to Page28, Line 523:

“Several studies have shown that HLA-DR can be used as a late activation marker for NK cells in various infectious diseases, with activated HLA-DR^+^NK cells peaking within 3 to 4 weeks after symptom onset and then declining. Additionally, CD69 is an early activation marker of NK cells, and CD69^+^CD56^dim^NK cells are significantly elevated in severe dengue patients even during recovery. In our study, we detected CD69 expression on CD56^dim^NKG2A^+^NK cells in PBMCs from 19 acute-phase and 7 convalescent-phase patients, aiming to further investigate the activation status of NK cells in the context of specific infections.”

References:

Ref 1. Tarantino N, Leboyer M, Bouleau A, Hamdani N, et al. Natural killer cells in first-episode psychosis: an innate immune signature? Mol Psychiatry. 2021;26(9):5297-5306.

Ref 2. N'guessan KF, Machmach K, Swafford I, Costanzo MC, et al. Innate immune cell activation after HIV-1 vaccine administration is associated with increased antibody production. Front Immunol. 2024;15:1339727.

Ref 3. Lee MJ, Blish CA. Defining the role of natural killer cells in COVID-19. Nat Immunol. 2023;24(10):1628-1638.

Ref 4. Koutsakos M, Rowntree LC, Hensen L, Chua BY, et al. Integrated immune dynamics define correlates of COVID-19 severity and antibody responses. Cell Rep Med. 2021;2(3):100208.

Ref 5. Ziegler SF, Ramsdell F, Alderson MR. The activation antigen CD69. Stem Cells. 1994;12(5):456-65.

Ref 6. Shabrish S, Karnik N, Gupta V, Bhate P, et al. Impaired NK cell activation during acute dengue virus infection: A contributing factor to disease severity. Heliyon. 2020;6(7):e04320.

Also, the author should explain the high expression of perforin, granzyme B, and CD107a in the uninfected control group, and the inconsistent expression profiles of TNF-a, Perforin, and CD107a in Fig.11 compared to Fig.6 and Fig.7.

**Response:** Thank you very much for your suggestions. We agree that it is essential to elucidate the high expression of perforin, granzyme B, and CD107a in the uninfected control group. Previous studies have demonstrated that healthy controls (HCs) exhibit high levels of granzyme B and perforin in total or CD56^dim^ NK cell populations, while CD107a expression is relatively low in total NK cells from HCs (Refs 1-4). Consistent with these findings, our study also observed high expression of perforin and granzyme B in CD56^dim^NKG2A^+^NKG2C^−^ NK cells from the PBMCs of uninfected controls. Additionally, studies have reported low CD107a expression in CD56^dim^NKG2A^+^NKG2C^−^ NK cells from PBMCs of HCMV-negative donors (Refs 5-6). Therefore, in the revised manuscript, we analyzed the degranulation levels of CD56^dim^NKG2A^+^ NK cells in fresh PBMCs from seven newly added uninfected controls. After incorporating these data, we found that CD56^dim^NKG2A^+^ NK cells in mild/moderate or severe/critical HFRS patients exhibited significantly higher CD107a expression compared to uninfected controls (*p*<0.05 and *p*<0.01, respectively). Furthermore, when comparing different disease stages, CD107a expression was elevated from the acute stage and remained at a higher level during the convalescent stage (*p*<0.01). Notably, CD107a expression on CD56^dim^NKG2A^+^ NK cells in HFRS patients at both stages was significantly higher than that in uninfected controls (*p*<0.05 and *p*<0.001, respectively).

The inconsistencies in the expression profiles of TNF-α, perforin, and CD107a observed in Figs 11, 6, and 7 can be attributed to the differences in the samples used for each analysis. Specifically, Figs 6 and 7 examined CD56^dim^NKG2A^+^ NK cells from PBMCs of HFRS patients and uninfected controls, whereas Fig 11 analyzed the expression of TNF-α, perforin, and CD107a on purified NK cells following co-culture with K562/HLA-E cells pulsed with HTNV-derived peptides (NP5). In fact, the *ex vivo* co-culture assay employed in Figure 11 serves as a valuable tool to investigate the impact of specific HTNV-derived peptides on the antiviral functions of CD56^dim^NKG2A^+^ NK cells. This approach further elucidates the mechanisms underlying the interaction between HLA-E/peptide complexes and the CD94/NKG2A receptor.

In the revised manuscript, the data described above have been incorporated into the Results section (Page 12, Line 247 to Page 15 Line 289) and are presented as a new figure (Figs 6 and 7).

Page 12, Line 247 to Page 15 Line 289:

“**CD56^dim^NKG2A^+^ NK Cells in HFRS Patients Correlate with Disease Severity and Clinical Course**

We investigated the production of TNF-α and IFN-γ by CD56^dim^NKG2A^+^NK cells in HFRS patients. The results showed that the frequencies of TNF-α^+^CD56^dim^NKG2A^+^NK cells were significantly higher in severe/critical and mild/moderate HFRS patients compared to uninfected controls (*p*<0.05 and *p*<0.05, respectively) (Figs 6A and B). Similarly, the levels of IFN-γ produced by CD56^dim^NKG2A^+^ NK cells were significantly elevated in both severe/critical and mild/moderate HFRS patients compared to uninfected controls (*p*<0.001 and *p*<0.05, respectively) (Figs 6C and D). Furthermore, during the acute and convalescent stages, HFRS patients exhibited higher frequencies of TNF-α^+^CD56^dim^NKG2A^+^ NK cells compared to uninfected controls (*p*<0.01 and *p*<0.001, respectively) (Figs 6G and H). Notably, the production of IFN-γ by CD56^dim^NKG2A^+^ NK cells was higher in HFRS patients during the acute stage compared to those in the convalescent stage and uninfected controls (*p*<0.05 and *p*<0.001, respectively) (Figs 6I and J). The frequency of TNF-α^+^IFN-γ^+^ CD56^dim^NKG2A^+^ NK cells was also higher during the acute stage compared to the convalescent stage and uninfected controls (*p*<0.05 and *p*<0.01, respectively) (Figs 6K and L), although no significant differences were observed between different disease severity groups (Figs 6E and F).

We further examined the production of cytotoxic mediator perforin and granzyme B, as well as the degranulation of CD56^dim^NKG2A^+^ NK cells. Although no significant differences were observed between HFRS patients and uninfected controls in the frequencies of perforin^+^ or granzyme B^+^CD56^dim^NKG2A^+^ NK cells (Figs 7A-D and G-J), the expression of CD107a was significantly higher in mild/moderate and severe/critical HFRS patients compared to uninfected controls (*p*<0.01 and *p*<0.001, respectively) (Figs 7E and F). When comparing different disease stages, CD107a expression was elevated during the acute stage and remained at higher levels during the convalescent stage (*p*<0.01). The frequency of CD107a^+^CD56^dim^NKG2A^+^ NK cells was also higher at both stages compared to uninfected controls (*p*<0.05 and *p*<0.001, respectively) (Figs 7G and H).”

References:

Ref 1. Malengier-Devlies B, Filtjens J, Ahmadzadeh K, Boeckx B, et al. Severe COVID-19 patients display hyper-activated NK cells and NK cell-platelet aggregates. Front Immunol. 2022;13:861251.

Ref 2. Du Y, Anastasiou OE, Strunz B, Scheuten J, et al. The impact of hepatitis B surface antigen on natural killer cells in patients with chronic hepatitis B virus infection. Liver Int. 2021;41(9):2046-2058.

Ref 3. Costanzo MC, Creegan M, Lal KG, Eller MA. OMIP-027: Functional analysis of human natural killer cells. Cytometry A. 2015; 87(9):803-5.

Ref 4. Liu S, Xu C, Yang F, Zong L, et al. Natural Killer Cells Induce CD8+ T Cell Dysfunction via Galectin-9/TIM-3 in Chronic Hepatitis B Virus Infection. Front Immunol. 2022;13:884290.

Ref 5. Hasan MZ, Claus M, Krüger N, Reusing S, et al. SARS-CoV-2 infection induces adaptive NK cell responses by spike protein-mediated induction of HLA-E expression. Emerg Microbes Infect. 2024;13(1):2361019.

Ref 6. d'Angelo P, Zavaglio F, Gabanti E, Zelini P, et al. Evaluation of the In Vitro Capacity of Anti-Human Cytomegalovirus Antibodies to Initiate Antibody-Dependent Cell Cytotoxicity. Microorganisms. 2024;12(7):1355.

Further, the statistical analysis of the increase of NK cell cytotoxic capacity in the presence of HTNV-derived peptide in Fig.13 need to be enhanced to support the conclusion.

**Response:** We sincerely appreciate your insightful suggestion. To strengthen the statistical analysis supporting the enhanced NK cell cytotoxicity in the presence of HTNV-derived peptides (Fig 13), we have expanded both the sample size (increasing the number of acute-phase HFRS patients and uninfected controls) and the diversity of control groups (including no-peptide, irrelevant peptide, and HLA leader peptide controls). This expansion significantly improved the statistical power of our comparative analyses (detailed in revised Fig 13).

Critically, our updated results confirm that CD56^dim^NKG2A^+^NK cells specifically exhibit heightened cytotoxicity against HTNV-GP8 peptide-pulsed targets (vs. HLA-Cw*03 leader peptide controls, *p* < 0.05 at 3:1 E:T ratio), whereas no such enhancement was observed for CD56^dim^NKG2A^−^NK cells or in groups stimulated with non-HTNV peptides (Figs 13B-C). Importantly, this GP8-specific activity aligns with our mechanistic findings that four identified HTNV-derived peptides (including GP8) fail to engage the CD94/NKG2A inhibitory axis, thereby bypassing NK cell suppression and enabling degranulation. In contrast, the HLA-Cw*03 leader peptide (VMAPRTLIL), which binds HLA-E and activates CD94/NKG2A-mediated inhibition (Refs 1-2), significantly dampened CD56^dim^NKG2A^+^NK cell killing (*p* < 0.05 vs. GP8). We think these further detection and analysis may support our conclusion that HTNV-derived peptides selectively enhance NK cell cytotoxicity by evading CD94/NKG2A-mediated inhibition.

In the revised manuscript, the data have been incorporated into a paragraph in the Results section (Page24, Line 423 to Line 434), and Fig 13 has been added to display the results. Additionally, a thorough discussion regarding the cytolytic capacity of CD56^dim^NKG2A^+^ NK cells has been included (Page 29, Line 604 to Line 615). The experiments are described in detail in the Materials and Methods section (Page 35, Line 850 to Page 36, Line 870).

Page24, Line 423 to Line 434:

“ To evaluate the specific cytotoxic capacity of CD56^dim^NKG2A^+^ NK cells against HTNV peptide-loaded targets, we conducted comparative cytotoxicity assays between CD56^dim^NKG2A^+^ and CD56^dim^NKG2A^−^NK cell subsets (Fig. 13A). Strikingly, at a 3:1 effector-to-target (E:T) ratio, CD56^dim^NKG2A^+^NK cells exhibited significantly enhanced killing activity in the GP8 peptide-stimulated group compared to the HLA-Cw*03 leader peptide control (*p*<0.05), as evidenced by elevated percentages of EGFP^+^Fixable Viability Dye eFluor 450^+^ dead cells (Fig. 13B). This GP8-specific enhancement, however, was not observed in CD56^dim^NKG2A^−^ NK cells. Importantly, neither subset demonstrated differential cytotoxicity between the HLA-Cw03 leader peptide control and groups lacking peptide stimulation, exposed to alternative control peptides, or treated with other HTNV-derived peptides (Figs. 13B and C), highlighting the selective response to GP8 peptide in CD56^dim^NKG2A^+^ NK cells.”

Page 29, Line 604 to Line 615:

“Furthermore, our findings collectively demonstrate that HLA-E-restricted presentation of the HTNV-GP8 peptide (MMKSCLIAL) selectively enhances the cytotoxic potential of CD56^dim^NKG2A^+^ NK cells by evading CD94/NKG2A-mediated inhibitory signaling. Notably, *in vitro* co-culture assays revealed that the HLA-E/HTNV-GP8 peptide complex significantly boosted the killing capacity of CD56^dim^NKG2A^+^NK cells against HLA-E-expressing targets (Fig. 13), a phenomenon not observed with control peptides such as the HLA-Cw*03 leader sequence (VMAPRTLIL) that actively engage the CD94/NKG2A axis. Specifically, the structural incompatibility between HTNV-derived peptides (including GP8) and the CD94/NKG2A receptor likely prevents inhibitory signaling, thereby "unshackling" CD56^dim^NKG2A^+^ NK cells to execute anti-viral cytotoxicity. These insights highlight the therapeutic potential of designing HLA-E-restricted peptides that selectively modulate NK cell responses against viral infections.”

Page 35, Line 850 to Page 36, Line 870:

“**Isolation of NK cells with FACS**

PBMCs (3×10⁷) from uninfected controls were stained with PE-Cy7-labeled anti-human CD3 mAb (Clone 17A2, BioLegend, USA), PE-labeled anti-human CD56 mAb (Clone 5.1H11, BioLegend, USA), and APC-labeled anti-human NKG2A mAb (Clone S19004C, BioLegend, USA). The cells were incubated for 30 minutes at 4°C in the dark. After incubation, the cells were washed and resuspended in RPMI-1640 medium (Gibco, USA). The CD3⁻CD56^dim^NKG2A^+^NK cells and CD3⁻CD56^dim^NKG2A⁻NK cells were sorted using a BD FACS Aria IIU system (BD Biosciences, USA). The sorted cells were then rested in complete RPMI-1640 medium supplemented with 200 U/ml IL-2 (MedChemExpress, USA) overnight at 37°C and 5% CO₂ before the start of the killing assay.

**NK Cell Cytotoxicity Assay**

The CD3⁻CD56^dim^NKG2A^+^NK cells and CD3⁻CD56^dim^NKG2A⁻NK cells, sorted by FACS from PBMCs of uninfected controls, were used as effector cells. EGFP-labeled K562/HLA-E cells pulsed with HTNV-derived peptides (NP5, GP7, GP8, and GP20) were used as target cells. The effector cells were co-cultured with 1×10⁴ peptide-loaded K562/HLA-E target cells in a 96-well U-bottom plate at an effector-to-target (E:T) ratio of 3:1. Control groups included NK cells only and EGFP-labeled K562/HLA-E cells only. After a 5-hour incubation at 37°C, cells were stained for viability using Fixable Viability Dye eFluor 450 (BioLegend, USA) at the manufacturer’s recommended concentration for 30 minutes at 4°C. The cells were then analyzed by flow cytometry using an ACEA NovoExpress system (Agilent Technologies, USA). EGFP^+^ Fixable Viability Dye eFluor 450^+^ cells were identified as killed target cells.”

Reference:

Ref 1. Tarragó D, González I, González-Escribano MF. HLA-E restricted cytomegalovirus UL40 peptide polymorphism may represent a risk factor following congenital infection. BMC Genomics. 2022;23(1):455.

Ref 2. Mbiribindi B, Pena JK, Arvedson MP, Moreno Romero C, et al. Epstein-Barr virus peptides derived from latent cycle proteins alter NKG2A + NK cell effector function. Sci Rep. 2020;10(1):19973.

2. The sample size is relatively small. This limits the statistical power of the study. The authors should clearly state the limitations in the discussion, and also tone down the conclusion about the correlations between NK cell characteristics and disease severity.

**Response:** We sincerely appreciate the editor’s critical suggestion regarding the sample size and the conclusion with an appropriate tone. In fact, according to the Chinese Center for Disease Control and Prevention, reported HFRS cases declined from 9,187 in 2021 to 5,218 in 2022, reflecting a nationwide reduction in disease incidence that complicated patient recruitment during our study period. Moreover, resource constraints precluded single-cell RNA sequencing (scRNA-Seq) of additional PBMC samples, which could have provided deeper mechanistic insights. We acknowledge the editor’s point as a critical direction for future work and have revised the Discussion section for the limitation.

To address the editor’s concern about overstating conclusions, we have revised the text to temper claims about disease severity correlations. For example, in the original conclusion stating "These results strongly indicate that NK cell dysfunction directly contributes to HFRS progression,*"* we have rephrased it to (Page 28, Line 554 to Line 556):
"These preliminary findings suggest a potential link between NK cell functional profiles and HFRS severity, though further validation in expanded cohorts is necessary to establish causality."

We further emphasize that our statistical analyses were designed to maximize rigor within the available sample size. Notably, the GP8 peptide-specific enhancement of CD56^dim^NKG2A^+^NK cell cytotoxicity remained statistically significant (*p* < 0.05) even after conservative adjustments, underscoring the robustness of this key finding.

In the revised manuscript, following the editor’s suggestion, we have updated the title in the Results section (Page 6, Line 144 to Line 146). Additionally, we have incorporated the discussion mentioned above to provide explanations regarding the small sample size of PBMCs from HFRS patients (Page 26, Line 494 to Line 498).

Page 6, Line 144 to Line 146:

“**Two distinct subclusters of NK cells exhibited high expression of NKG2A and displayed signatures associated with inflammation and proliferation during HTNV infection**”

Page 26, Line 494 to Line 498:

“The sample size of HFRS patients, though aligned with recent epidemiological trends in China, remains relatively small, which is an obvious limitation for this study. While our findings suggest associations between NK cell characteristics and disease severity, these observations should be interpreted as preliminary and require future validation in larger, multi-center cohorts.”

3. As the suggestion by two reviewers, to explain the specific effect of peptide NP5, a peptide known to induce an inhibitory effect on NK cells may be needed.

**Response:** We sincerely thank the editor and reviewers for raising this critical point regarding the functional characterization of the NP5 peptide. As requested, we have now incorporated comprehensive analyses of all four HTNV-derived peptides (NP5, GP7, GP8, and GP20) into the revised manuscript to clarify their distinct effects on NK cell activity. Below, we address the specific role of NP5 in detail:

First, NP5 exhibits high HLA-E binding affinity but fails to engage CD94/NKG2A inhibition. In our HLA-E*0103 binding assays, NP5 demonstrated the highest binding affinity among the four HTNV-derived peptides (fluorescence intensity [FI] = 2.20, Table 2). Despite this strong HLA-E interaction, NP5 did not suppress CD56^dim^NKG2A^+^NK cell cytotoxicity in functional assays (revised Figs 13A-B). This contrasts with the HLA-Cw*03 leader peptide (VMAPRTLIL), which robustly inhibited NK cell activity via CD94/NKG2A engagement. The dissociation between HLA-E binding and inhibitory signaling suggests that NP5 lacks structural complementarity to the CD94/NKG2A receptor, a prerequisite for initiating inhibitory signaling (Ref 1).

Second, due to initial funding limitations, NP5 was prioritized for early experiments based on its high HLA-E binding capacity (Table 2) and its reported immunogenicity in CD8^+^ T cell responses (Ref 5). In the revised manuscript, we have now expanded all functional assays to include all four peptides (NP5, GP7, GP8, GP20). These data are presented in revised Figs 12-13 and described in the Results section (Page 22, Line 399 to Page 25, Line 434).

We thank the reviewers for prompting this deeper exploration, which has strengthened the mechanistic clarity of our findings. Future structural studies such as HLA-E/NP5/NKG2A co-crystallization may be warranted to validate the effects of HLA-E/HTNV-NP5 for CD56^dim^NKG2A^+^ NK cells. We have added this content to the Discussion section in the revised manuscript (Page 29, Line 616 to Line 621).

Page 22, Line 399 to Page 25, Line 434:

“To further elucidate the impact of peptides presented by HLA-E on the function of CD56^dim^NKG2A^+^NK cells, we analyzed the functional phenotypes and indicators across different peptide groups. Our results demonstrated that CD56^dim^NKG2A^+^NK cells from uninfected controls, when co-cultured with K562/HLA-E cells loaded with HTNV peptides, exhibited significantly higher levels of CD107a expression compared to those co-cultured with cells presenting the HLA-Cw*03 leader peptide control (*p*<0.05 and *p*<0.01, respectively) (Figs 12A and B). Moreover, we compared the degranulation activity between CD56^dim^NKG2A^+^ and CD56^dim^NKG2A^−^NK cells. In contrast, CD56^dim^NKG2A^−^ NK cells did not exhibit significant differences in degranulation across the various groups (Figs 12C and D). Additionally, the expression of perforin and TNF-α in both CD56^dim^NKG2A^+^ and CD56^dim^NKG2A^−^ NK cells remained unchanged across all groups (Figs 12E-L).”

**The HLA-E/HTNV-derived peptide complex enhanced the cytotoxic capacity of CD56^dim^NKG2A^+^NK cells at the acute stage of HFRS**

To evaluate the specific cytotoxic capacity of CD56^dim^NKG2A^+^NK cells against HTNV peptide-loaded targets, we conducted comparative cytotoxicity assays between CD56^dim^NKG2A^+^ and CD56^dim^NKG2A^−^NK cell subsets (Fig 13A). Strikingly, at a 3:1 effector-to-target (E:T) ratio, CD56^dim^NKG2A^+^NK cells exhibited significantly enhanced killing activity in the GP8 peptide-stimulated group compared to the HLA-Cw*03 leader peptide control (*p*<0.05), as evidenced by elevated percentages of EGFP^+^Fixable Viability Dye eFluor™ 450^+^ dead cells (Fig 13B). This GP8-specific enhancement, however, was not observed in CD56^dim^NKG2A^−^NK cells. Importantly, neither subset demonstrated differential cytotoxicity between the HLA-Cw*03 leader peptide control and groups lacking peptide stimulation, exposed to alternative control peptides, or treated with other HTNV-derived peptides (Figs 13B and C), highlighting the selective response to GP8 peptide in CD56^dim^NKG2A^+^ NK cells.

Page 29, Line 616 to Line 621:

Notably, the HTNV-NP5 peptide exhibited strong HLA-E*0103 binding (FI=2.20) yet failed to engage CD94/NKG2A-mediated NK cell inhibition (Figs. 13B-C). This functional-structural dissociation suggests NP5 may potentially compete with inhibitory peptides to enhance NK cell surveillance while retaining immunogenicity for CD8^+^ T cell activation. Structural characterization of the HLA-E/NP5/NKG2A interface is warranted to resolve this mechanism in future. ”

**Journal Requirements:**

1) Please ensure that the CRediT author contributions listed for every co-author are completed accurately and in full.
At this stage, the following Authors/Authors require contributions: Xue Manling, Tang Kang, Zhang Yusi, Xu Xiaoyue, Zhang Chunmei, Zuo Jiajia, Wang Fenglan, Zhang Xiyue, Zheng Xuyang, Zhuang Ran, Zhang Yun, Jin Boquan, and Ying Ma. Please ensure that the full contributions of each author are acknowledged in the "Add/Edit/Remove Authors" section of our submission form.
The list of CRediT author contributions may be found here: https://journals.plos.org/plospathogens/s/authorship#loc-author-contributions

**Response:** Thank you for pointing out this error. We have reviewed and corrected the Author Contributions in the CRediT author contributions list (Page 36, Line 882 to Line 896). The revised author contributions have been updated and are now accurately reflected in the “Author Contributions” section.

Page 36, Line 882 to Line 896:

“**Author Contributions**

**Conceptualization**: Ying Ma.

**Data Curation**: Kang Tang, Yusi Zhang.

**Formal Analysis**: Manling Xue, Xiaoyue Xu, Jiajia Zuo.

**Funding Acquisition**: Ying Ma, Kang Tang, Yusi Zhang, Chunmei Zhang, Yun Zhang.

**Investigation**: Chunmei Zhang.

**Methodology**: Ying Ma, Manling Xue.

**Project Administration**: Ying Ma.

**Resources**: Ying Ma.

**Software**: Fenglan Wang.

**Supervision**: Ran Zhuang, Yun Zhang.

**Validation**: Xiyue Zhang.

**Visualization**: Xuyang Zheng.

**Writing-Original Draft Preparation**: Manling Xue.

**Writing-Review & Editing**: Ying Ma, Boquan Jin.”

2) We note that your Manuscript "Hantaan virus-derived peptides.pdf" and Manuscript " Manuscript.pdf" files are duplicated on your submission. Please remove any unnecessary or old files from your revision, and make sure that only those relevant to the current version of the manuscript are included.

**Response:** We are grateful to you for pointing out this error. The unnecessary file "Hantaan virus-derived peptides.pdf" has been removed from the revision.

3) We ask that a manuscript source file is provided at Revision. Please upload your manuscript file as a .doc, .docx, .rtf or .tex. If you are providing a .tex file, please upload it under the item type u2018LaTeX Source Fileu2019 and leave your .pdf version as the item type u2018Manuscriptu2019.

**Response:** Thank you for your reminder. We have submitted the following three documents through the system: "Response to Reviewers," "Revised Manuscript with Track Changes," and "Manuscript."

4) Please ensure that the funders and grant numbers match between the Financial Disclosure field and the Funding Information tab in your submission form. Note that the funders must be provided in the same order in both places as well. State what role the funders took in the study. If the funders had no role in your study, please state: "The funders had no role in study design, data collection and analysis, decision to publish, or preparation of the manuscript."
If you did not receive any funding for this study, please simply state: u201cThe authors received no specific funding for this work.u201d

**Response:** Thank you for your reminder. We have matched the funders and grant numbers between the Financial Disclosure field and the Funding Information tab in my submission form.

**Reviewers' Comments:**

Reviewer's Responses to Questions

**Part I - Summary**
Please use this section to discuss strengths/weaknesses of study, novelty/significance, general execution and scholarship.

Reviewer #1: Xue et al investigated the NK cell responses in HFRS patients cause by Hantaan virus (HTNV) infection. They described that several HTNV-derived peptides can stabilize HLA-E expression on the target cells, but the HLA-E/HTNV-derived peptide complex are not recognized by CD94/NKG2A receptor on NK cells, which may enhance the NK cell activation during the virus infections. However, the increase of NK cell cytotoxic capacity in the presence of HTNV-derived peptide are neglectable. Many of the data in this manuscript have inconsistencies with each other.

**Response:** Thank you for your affirmation and evaluation of the research in this manuscript. Indeed, the statistical analysis of the increase of NK cell cytotoxic capacity in the presence of HTNV-derived peptide need to be enhanced to support our conclusion. Meanwhile, as mentioned by editor, there were inconsistent expression profiles of TNF-a, Perforin, and CD107a in Fig 11 compared to Fig 6 and Fig7.

To strengthen the statistical support for HTNV-derived peptide-enhanced NK cell cytotoxicity (Fig 13), we have expanded both the cohort size (increasing acute-phase HFRS patients and uninfected controls) and the diversity of control groups (no-peptide, irrelevant peptide, and HLA leader peptide controls). These revisions significantly improved the statistical robustness of our analyses (revised Fig 13). Critically, our updated data confirm that CD56^dim^NKG2A^+^NK cells selectively exhibit heightened cytotoxicity against HTNV-GP8 peptide-pulsed targets (vs. HLA-Cw*03 leader peptide controls, *p* < 0.05 at 3:1 E:T ratio), whereas no such enhancement occurred in CD56^dim^NKG2A^−^NK cells or with non-HTNV peptides (Figs 13B-C). This GP8-specific activity aligns mechanistically with our finding that four HTNV-derived peptides (including GP8) evade CD94/NKG2A-mediated inhibition, thereby permitting NK cell degranulation. In contrast, the HLA-Cw*03 leader peptide (VMAPRTLIL), which binds HLA-E to activate CD94/NKG2A-dependent suppression (Ref. 1), significantly reduced CD56^dim^NKG2A^+^ NK cell cytotoxicity (*p* < 0.05 vs. GP8). Together, these results could support our conclusion that HTNV peptides selectively enhance NK cell cytotoxicity by bypassing CD94/NKG2A inhibitory signaling. The revised manuscript now incorporates these data into the Results section (Page24, Line 423 to Line 434), with detailed experimental methods provided in Materials and Methods (Page 35, Line 850 to Page 36, Line 870). A dedicated discussion of CD56^dim^NKG2A^+^ NK cell cytolytic activity has also been added (Page 29, Line 604 to Line 615).

The discrepancies in the expression profiles of TNF-α, perforin, and CD107a observed in Figs 11, 6, and 7 can be attributed to the differences in the samples used for each analysis. Specifically, Figures 6 and 7 examined CD56^dim^NKG2A^+^ NK cells from PBMCs of HFRS patients and uninfected controls, while Figure 11 analyzed the expression of TNF-α, perforin, and CD107a on purified NK cells after co-culture with K562/HLA-E cells pulsed with HTNV-derived peptides (NP5). The *ex vivo* co-culture assay used in Fig 11 may be a valuable tool for investigating the impact of specific HTNV-derived peptides on the antiviral functions of CD56^dim^NKG2A^+^ NK cells. This approach further elucidates the mechanisms underlying the interaction between HLA-E/peptide complexes and the CD94/NKG2A receptor. In the revised manuscript, these data have been incorporated and are presented as a new figure (Figs 7A-D).

Page24, Line 423 to Line 434:

“ To evaluate the specific cytotoxic capacity of CD56^dim^NKG2A^+^ NK cells against HTNV peptide-loaded targets, we conducted comparative cytotoxicity assays between CD56^dim^NKG2A^+^ and CD56^dim^NKG2A^−^ NK cell subsets (Fig 13A). Strikingly, at a 3:1 effector-to-target (E:T) ratio, CD56^dim^NKG2A^+^ NK cells exhibited significantly enhanced killing activity in the GP8 peptide-stimulated group compared to the HLA-Cw*03 leader peptide control (*p*<0.05), as evidenced by elevated percentages of EGFP^+^Fixable Viability Dye eFluor 450^+^ dead cells (Fig 13B). This GP8-specific enhancement, however, was not observed in CD56^dim^NKG2A^−^ NK cells. Importantly, neither subset demonstrated differential cytotoxicity between the HLA-Cw*03 leader peptide control and groups lacking peptide stimulation, exposed to alternative control peptides, or treated with other HTNV-derived peptides (Figs 13B and C), highlighting the selective response to GP8 peptide in CD56^dim^NKG2A^+^ NK cells.”

Page 29, Line 604 to Line 615:

“Furthermore, our findings collectively demonstrate that HLA-E-restricted presentation of the HTNV-GP8 peptide (MMKSCLIAL) selectively enhances the cytotoxic potential of CD56^dim^NKG2A^+^ NK cells by evading CD94/NKG2A-mediated inhibitory signaling. Notably, in vitro co-culture assays revealed that the HLA-E/HTNV-GP8 peptide complex significantly boosted the killing capacity of CD56^dim^NKG2A^+^ NK cells against HLA-E-expressing targets (Fig. 13), a phenomenon not observed with control peptides such as the HLA-Cw*03 leader sequence (VMAPRTLIL) that actively engage the CD94/NKG2A axis. Specifically, the structural incompatibility between HTNV-derived peptides (including GP8) and the CD94/NKG2A receptor likely prevents inhibitory signaling, thereby "unshackling" CD56^dim^NKG2A^+^ NK cells to execute anti-viral cytotoxicity. These insights highlight the therapeutic potential of designing HLA-E-restricted peptides that selectively modulate NK cell responses against viral infections.”

Page 35, Line 850 to Page 36, Line 870:

“**Isolation of NK cells with FACS**

PBMCs (3×10⁷) from uninfected controls were stained with PE-Cy7-labeled anti-human CD3 mAb (Clone 17A2, BioLegend, USA), PE-labeled anti-human CD56 mAb (Clone 5.1H11, BioLegend, USA), and APC-labeled anti-human NKG2A mAb (Clone S19004C, BioLegend, USA). The cells were incubated for 30 minutes at 4°C in the dark. After incubation, the cells were washed and resuspended in RPMI-1640 medium (Gibco, USA). The CD3⁻CD56^dim^NKG2A^+^ NK cells and CD3⁻CD56^dim^NKG2A⁻ NK cells were sorted using a BD FACSAria™ IIU system (BD Biosciences, USA). The sorted cells were then rested in complete RPMI-1640 medium supplemented with 200 U/ml IL-2 (MedChemExpress, USA) overnight at 37°C and 5% CO₂ before the start of the killing assay.

**NK Cell Cytotoxicity Assay**

The CD3⁻CD56^dim^NKG2A^+^ NK cells and CD3⁻CD56^dim^NKG2A⁻ NK cells, sorted by FACS from PBMCs of uninfected controls, were used as effector cells. EGFP-labeled K562/HLA-E cells pulsed with HTNV-derived peptides (NP5, GP7, GP8, and GP20) were used as target cells. The effector cells were co-cultured with 1×10⁴ peptide-loaded K562/HLA-E target cells in a 96-well U-bottom plate at an effector-to-target (E:T) ratio of 3:1. Control groups included NK cells only and EGFP-labeled K562/HLA-E cells only. After a 5-hour incubation at 37°C, cells were stained for viability using Fixable Viability Dye eFluor™ 450 (BioLegend, USA) at the manufacturer’s recommended concentration for 30 minutes at 4°C. The cells were then analyzed by flow cytometry using an ACEA NovoExpress system (Agilent Technologies, USA). EGFP^+^ Fixable Viability Dye eFluor™ 450^+^ cells were identified as killed target cells.”

Reference:

1.Tarragó D, González I, González-Escribano MF. HLA-E restricted cytomegalovirus UL40 peptide polymorphism may represent a risk factor following congenital infection. BMC Genomics. 2022;23(1):455.

Mbiribindi B, Pena JK, Arvedson MP, Moreno Romero C, McCarthy SR, Hatton OL, Esquivel CO, Martinez OM, 2.Krams SM. Epstein-Barr virus peptides derived from latent cycle proteins alter NKG2A + NK cell effector function. Sci Rep. 2020;10(1):19973.

Reviewer #2: This manuscript, titled "Hantaan Virus-Derived Peptides That Stabilize HLA-E Could Abrogate Inhibition of CD56dimNKG2A+ NK Cells," investigates the role of Hantaan virus (HTNV)-derived peptides in modulating the activity of CD56dimNKG2A+ natural killer (NK) cells in patients with hemorrhagic fever with renal syndrome (HFRS). The study employs a combination of single-cell RNA sequencing, flow cytometry, and functional assays to characterize NK cell subsets and thoroughly examines their responses to HTNV peptides. The findings indicate that specific HTNV peptides presented by HLA-E can counteract the inhibitory effect of NKG2A, thereby enhancing antiviral immunity. Additionally, the study offers valuable mechanistic insights into how HTNV-derived peptides may evade NK cell-mediated inhibition, which could inform future therapeutic developments. While the study delivers intriguing results and demonstrates strong methodology, further clarification and improvements are required.

In summary, this study offers valuable insights into the interactions among HTNV, HLA-E, and NK cells. However, it has notable limitations, including a very small sample size, the necessity for additional experiments to confirm the observed correlations, and a lack of definitive mechanistic details. Addressing these concerns will significantly enhance the manuscript's robustness and increase its overall impact.

**Response:** We sincerely thank the reviewer for their thoughtful summary and constructive critique of our work. We fully acknowledge the study’s limitations and have discussed in the revised manuscript to address these concerns. According to the Chinese Center for Disease Control and Prevention, the number of reported HFRS cases decreased from 9,187 in 2021 to 5,218 in 2022. This nationwide reduction in disease incidence significantly complicated patient recruitment during our study period. Although our study provides foundational insights into HLA-E/NK cell/HTNV interactions, we agree that larger cohorts are needed for future study.

To address the need for deeper mechanistic insights, we added the experiments to demonstrated that HTNV-derived peptides (including GP8) fail to engage the CD94/NKG2A inhibitory axis, enabling NK cell degranulation (Figs. 12-13). In contrast to GP8, the HLA-Cw03 leader peptide (VMAPRTLIL) binds HLA-E to trigger CD94/NKG2A-mediated inhibitory signaling, thereby suppressing NK cell activity. Meanwhile, we have expanded our analyses to include all HTNV-derived peptides (NP5, GP7, GP8, GP20) in Figures 12-13, consolidated *ex vivo* PBMC data and *in vitro* co-culture assays, and strengthened the Discussion with mechanistic insights into peptide-specific effects. Furthermore, we emphasized several future directions in the Discussion section. For example, co-crystallization of HLA-E/NP5/NKG2A complexes is warranted to resolve the molecular basis for NP5 of inability to trigger inhibitory signaling. For multi-omics integration, longitudinal multi-center cohorts will refine our understanding of NK cell dynamics during HFRS progression.

Based on these, we incorporated the discussion mentioned above to provide explanations regarding the small sample size of PBMCs from HFRS patients (Page 26, Line 494 to Line 498) and mechanistic explanations (Page 29, Line 616 to Line 621) in the revised manuscript.

Page 26, Line 494 to Line 498:

“The sample size of HFRS patients, though aligned with recent epidemiological trends in China, remains relatively small, which is an obvious limitation for this study. While our findings suggest associations between NK cell characteristics and disease severity, these observations should be interpreted as preliminary and require future validation in larger, multi-center cohorts.”

Page 29, Line 616 to Line 621:

“Notably, the HTNV-NP5 peptide exhibited strong HLA-E*0103 binding (FI=2.20) yet failed to engage CD94/NKG2A-mediated NK cell inhibition (Figs. 13B-C). This functional-structural dissociation suggests NP5 may potentially compete with inhibitory peptides to enhance NK cell surveillance while retaining immunogenicity for CD8^+^ T cell activation. Structural characterization of the HLA-E/NP5/NKG2A interface is warranted to resolve this mechanism in future. ”

Reviewer #3: The authors investigated the role of NK cells in Hantaan virus induced hemorrhagic fever with renal syndrome (HFRS). NK subtypes in HFRS patients were quantified and none of 4 virus peptides that could be presented by HLA-E was recognized by NKG2A, suggesting that a lack of recognition might leading to enhanced NK cell activity by not triggering the NK cell inhibitory activity mediated by NKG2A. The results are somewhat convincing but strictly correlative.

**Response:** We sincerely appreciate the reviewer’s comments. We agree that the results are somewhat convincing but strictly correlative, including the conclusion that the increase of NK cell cytotoxic capacity in the presence of HTNV-derived peptide, as suggested by the Reviewer 1. While our study provides valuable insights into the activation and cytotoxic capacity of NK cells in HFRS, further mechanistic studies are needed to fully elucidate the underlying pathways. Specifically, we have identified that none of the four HTNV-derived peptides (NP5, GP7, GP8, GP20) are recognized by the NKG2A receptor, suggesting that the lack of inhibitory signaling through NKG2A leads to enhanced NK cell activity. This finding is consistent with previous studies demonstrating the critical role of NK cells in the immune response to HFRS.

To address the limitations of our current study and build on our findings, we propose several future directions. First, co-crystallization of HLA-E/NP5/NKG2A complexes is warranted to resolve the molecular basis for NP5’s inability to trigger inhibitory signaling. This structural analysis will provide a deeper understanding of the interactions between viral peptides and the NKG2A receptor. Second, longitudinal multi-center cohorts combined with single-cell RNA sequencing (scRNA-Seq) and spatial transcriptomics will refine our understanding of NK cell dynamics during HFRS progression.

In order to make more convincing about the results, we have made several key revisions to the manuscript. We redesigned the cytotoxicity assay for NK cells and consolidated the *ex vivo* PBMC data and i*n vitro* co-culture assays into a revised Fig 12 for direct comparison. Additionally, we have added detailed mechanistic explanations for peptide-specific effects and addressed the limitations of our study in the Discussion section.

**Part II – Major Issues: Key Experiments Required for Acceptance**
Please use this section to detail the key new experiments or modifications of existing experiments that should be absolutely required to validate study conclusions.

Generally, there should be no more than 3 such required experiments or major modifications for a "Major Revision" recommendation. If more than 3 experiments are necessary to validate the study conclusions, then you are encouraged to recommend "Reject".

Reviewer #1: 1. It seems that the 0 cluster of NK cells are the most abundant subsets of NK cells in HFRS patients, which express lower level of NKG2A, CD16 and CD56. What is the function and activation status of the cluster 0 in HFRS patients?

**Response:** Thank you for pointing out this important issue. We agree that a clearer explanation for the function and activation status of the cluster 0 of NK cells in HFRS patients is needed. The cluster 0, being the most abundant subset of NK cells in HFRS patients, exhibits relatively low expression of genes encoding *NCAM1* (CD56), *FCGR3A* (CD16), *KLRC1* (NKG2A), *KLRC2* (NKG2C), and *B3GAT1* (CD57). This suggests that the cluster 0 primarily presents a phenotype of CD56^-/low^CD16^low^NKG2A^low^NKG2C^low^ CD57^low^.

In healthy individuals, the CD56^-^ NK cell subset is a minor component of the peripheral blood NK cell compartment (ref 1). However, in chronic viral infections such as HIV-1 and HCV, CD56^-^ NK cells can expand significantly and constitute up to half of all peripheral NK cells (ref 2). These expanded CD56^-^ NK cells share a phenotype of CD56^-^CD16^low/+^NKG2A^low^NKG2C^low^CD57^low^, which is similar to the cluster 0 observed in our study (ref 2). Importantly, CD56^-^ NK cells in chronic infections often display an exhausted phenotype, characterized by reduced levels of perforin, granzyme B, IFN-γ, and TNF, as well as lower cytotoxic capacity compared to CD56^dim^ NK cells (refs 3-4).

In the context of HFRS, the low expression of key activation markers and cytotoxic molecules on cluster 0 suggests a potential state of functional exhaustion or an immature state. This phenotype may reflect a response to the prolonged or intense viral infection, where NK cells are unable to fully activate due to the lack of appropriate signals or the presence of inhibitory signals. Future studies should focus on characterizing the functional capacity and activation status of this subset in more detail, potentially through longitudinal analyses and functional assays, to better understand their role in HFRS pathogenesis and immune response.

References:

Ref 1. Mavilio D, Lombardo G, Benjamin J, Kim D, et al. Characterization of CD56-/CD16+ natural killer (NK) cells: a highly dysfunctional NK subset expanded in HIV-infected viremic individuals. Proc Natl Acad Sci U S A. 2005;102(8):2886-91.

Ref 2. Björkström NK, Ljunggren HG, Sandberg JK. CD56 negative NK cells: origin, function, and role in chronic viral disease. Trends Immunol. 2010;31(11):401-6.

Ref 3. Alter G, Teigen N, Davis BT, Addo MM, et al. Sequential deregulation of NK cell subset distribution and function starting in acute HIV-1 infection. Blood. 2005;106(10):3366-9.

Ref 4. Gonzalez VD, Falconer K, Björkström NK, Blom KG, et al. Expansion of functionally skewed CD56-negative NK cells in chronic hepatitis C virus infection: correlation with outcome of pegylated IFN-alpha and ribavirin treatment. J Immunol. 2009;183(10):6612-8.

2. In Figure 4, it is unusual to detect MHC class II molecular HLA-DR expression in NK cells. It is also unusual to detect high CD69 expression in NK cells of HFRS patients at convalescence stage. The author should explain this.

**Response:** Thank you for raising these important points regarding the unusual detection of HLA-DR expression in NK cells and high CD69 expression in NK cells during the convalescence stage of HFRS, as mentioned by the editor.

HLA-DR expression in NK cells has been observed in various infectious and inflammatory conditions (refs 1-3). For example, studies have shown that HLA-DR-positive NK cells can be found in healthy donors and are significantly increased in conditions such as HIV infection, tuberculosis, and COVID-19 (ref 4). Additionally, HLA-DR expression on NK cells has been associated with enhanced cytokine production and cytotoxic activity, making it a marker of activation. In the context of HFRS, the upregulation of HLA-DR on NK cells may reflect a heightened state of activation in response to the viral infection.

CD69 is an early activation marker typically expressed on activated NK cells (ref 5). While it is unusual to observe high CD69 expression during the convalescence stage, this phenomenon has been documented in other viral infections, such as Dengue fever, where CD69^+^ NK cells remain elevated even during recovery (ref 6). This sustained expression of CD69 may indicate a prolonged activation state or a slower resolution of the immune response in HFRS patients. In our study, we detected high CD69 expression in CD56^dim^NKG2A^+^ NK cells from both acute and convalescent HFRS patients, suggesting that these cells remain activated throughout the disease course.

In the revised manuscript, we have addressed these observations by providing a clearer analysis of the proportion of CD69^+^CD56^dim^NKG2A^+^ NK cells in PBMCs from HFRS patients at different disease stages and uninfected controls. The data have been added to the Results section (Page 10, Line 208 to Line 218) and presented as a new figure (Fig 4). Additionally, we have included a detailed discussion on the unusual detection of HLA-DR expression and high CD69 expression in NK cells during convalescence (Page27, Line 516 to Line 523).

Page 10, Line 208 to Line 218:

“Next, the activation status of CD56^dim^NKG2A^+^NK cells in HFRS patients were detected. Notably, the frequencies of HLA-DR^+^CD56^dim^NKG2A^+^NK cells in both mild/moderate and severe/critical HFRS patients were significantly higher than that in uninfected controls (*p*<0.01) (Figs 4A and B). The frequencies of CD69^+^CD56^dim^NKG2A^+^NK cells seem to be higher in both mild/moderate and severe/critical HFRS patients than that in uninfected controls, but showed no statistical difference among them. When comparing patients at different stages of the disease, the frequency of CD69^+^CD56^dim^NKG2A^+^NK cells was especially higher at convalescent stage than that at acute stage (p<0.01) (Figs 4C and D). The frequencies of both HLA-DR^+^CD56^dim^NKG2A^+^NK cells and CD69^+^CD56^dim^NKG2A^+^NK cells were higher at acute stage or convalescent stage of HFRS patients than that in uninfected controls (*p*<0.001 and *p*<0.001, respectively).”

Page27, Line 516 to Line 523:

“Several studies have shown that HLA-DR can be used as a late activation marker for NK cells in various infectious diseases, with activated HLA-DR^+^ NK cells peaking within 3 to 4 weeks after symptom onset and then declining. Additionally, CD69 is an early activation marker of NK cells, and CD69^+^CD56^dim^ NK cells are significantly elevated in severe dengue patients even during recovery. In our study, we detected CD69 expression on CD56^dim^NKG2A^+^ NK cells in PBMCs from 19 acute-phase and 7 convalescent-phase patients, aiming to further investigate the activation status of NK cells in the context of specific infections.”

References:

Ref 1. Tarantino N, Leboyer M, Bouleau A, Hamdani N, et al. Natural killer cells in first-episode psychosis: an innate immune signature? Mol Psychiatry. 2021;26(9):5297-5306.

Ref 2. N'guessan KF, Machmach K, Swafford I, Costanzo MC, et al. Innate immune cell activation after HIV-1 vaccine administration is associated with increased antibody production. Front Immunol. 2024;15:1339727.

Ref 3. Lee MJ, Blish CA. Defining the role of natural killer cells in COVID-19. Nat Immunol. 2023;24(10):1628-1638.

Ref 4. Koutsakos M, Rowntree LC, Hensen L, Chua BY, et al. Integrated immune dynamics define correlates of COVID-19 severity and antibody responses. Cell Rep Med. 2021;2(3):100208.

Ref 5. Ziegler SF, Ramsdell F, Alderson MR. The activation antigen CD69. Stem Cells. 1994;12(5):456-65.

Ref 6. Shabrish S, Karnik N, Gupta V, Bhate P, et al. Impaired NK cell activation during acute dengue virus infection: A contributing factor to disease severity. Heliyon. 2020;6(7):e04320.

3. In Figure 7, The trends of perforin, granzyme B, and CD107a in the same cells were not consistent. Granzyme B and CD107a should have similar trends under normal circumstances.

**Response:** Thank you for the suggestion to help us explain the correlation between granzyme B and CD107a. It is indeed unusual to observe inconsistent trends between granzyme B and CD107a, as both are typically associated with NK cell activation and cytotoxicity. However, several studies have shown that the expression of these markers can vary depending on the context of infection and the specific NK cell subset being examined. For example, in chronic hepatitis B infection, CD56^dim^ NK cells exhibited lower expression of perforin, granzyme B, and CD107a compared to healthy controls (Ref 1). This suggests that the expression of these markers can be influenced by the nature of the infection and the stage of disease. Additionally, in other viral infections such as COVID-19, CD56^dim^ NK cells showed variable expression of perforin and granzyme B, with some subsets displaying higher levels of granzyme B but not necessarily CD107a. This indicates that granzyme B and CD107a may not always follow the same trend, especially in complex disease settings (Ref 2).

In our study, we observed high levels of granzyme B but relatively lower CD107a expression in CD56^dim^NKG2A^+^ NK cells from uninfected controls compared to HFRS patients at the acute and convalescent phases. This deviation from the expected similar trends under normal conditions may reflect the unique activation status of NK cells in HFRS. The inconsistent trends could be due to differential regulation of these markers by cytokines or other immune signals present during HFRS infection.

References:

Ref 1. Liu S, Xu C, Yang F, Zong L, et al. Natural Killer Cells Induce CD8+ T Cell Dysfunction via Galectin-9/TIM-3 in Chronic Hepatitis B Virus Infection. Front Immunol. 2022;13:884290.

Ref 2. Malengier-Devlies B, Filtjens J, Ahmadzadeh K, Boeckx B, , et al. Severe COVID-19 patients display hyper-activated NK cells and NK cell-platelet aggregates. Front Immunol. 2022;13:861251.

4. Surprisingly, in the uninfected control group, the expression of perforin, granzyme B, and CD107a was so high (Fig. 7), the authors should use CD56dimCD16+NKG2A- NK cells as a control to compared the expression profiles of perforin, granzyme B, and CD107a between NKG2A+ and NKG2A- NK cells.

**Response:** We appreciated the reviewer’s suggestion, and we completely agree that it would be beneficial to use CD56^dim^CD16^+^NKG2A^-^NK cells as a control to make our conclusion much stronger. In the original manuscript, we observed that CD56^dim^NKG2A^+^ NK cells in uninfected controls had relatively high expression of perforin and granzyme B, but significantly lower levels of CD107a compared to HFRS patients at different disease stages. Following your suggestion, we have now compared the expression profiles of perforin, granzyme B, and CD107a between NKG2A^+^ and NKG2A^−^ NK cells using both existing data and new data from seven additional uninfected controls. Our analysis revealed no significant differences in the expression of these markers between NKG2A^+^ and NKG2A^−^ NK cells in uninfected controls.

To provide a more comprehensive view, we have included these results as supplementary data (please see Figure S3 for cytotoxic mediators and CD107a detection) in the revised manuscript. The details of this analysis have been added to the Results section (Page 15, Line 275 to Line 288). We believe that these additional analyses and the inclusion of CD56^dim^CD16^+^NKG2A^−^ NK cells as a control provide a stronger basis for our conclusions regarding the expression profiles of perforin, granzyme B, and CD107a in HFRS patients.

Page 15, Line 275 to Line 288:

“We further examined the production of cytotoxic mediator perforin and granzyme B, as well as the degranulation of CD56^dim^NKG2A^+^ NK cells. Although no significant differences were observed between HFRS patients and uninfected controls in the frequencies of granzyme B^+^ or perforin^+^ CD56^dim^NKG2A^+^ NK cells (Figs 7A-D and 7G-J), the expression of CD107a was significantly higher in mild/moderate and severe/critical HFRS patients compared to uninfected controls (*p*<0.01 and *p*<0.001, respectively) (Figs 7E and F). When comparing different disease stages, CD107a expression was elevated during the acute stage and remained at higher levels during the convalescent stage (*p*<0.01). The frequency of CD107a^+^CD56^dim^NKG2A^+^ NK cells was also higher at both stages compared to uninfected controls (*p*<0.05 and *p*<0.001, respectively) (Figs 7G and H).

Moreover, we compared the expression profiles of perforin, granzyme B, and CD107a between NKG2A^+^ and NKG2A^−^ NK cells using CD56^dim^CD16^+^NKG2A^−^ NK cells as a control. Our analysis revealed no significant differences in the expression of these markers between NKG2A^+^ and NKG2A^−^ NK cells in uninfected controls (Figs S3A and B).”

5. The expression profiles of TNF-a, Perforin, and CD107a in Fig.11 were not consistent with that in Fig.6 and Fig.7.

**Response:** Thank you very much for your careful review and for pointing out the inconsistencies in the expression profiles of TNF-α, perforin, and CD107a in Figures 11, 6, and 7. We appreciate your attention to this important aspect of our study.

The discrepancies in the expression profiles of TNF-α, perforin, and CD107a can be attributed to the differences in the samples and experimental conditions used for each analysis. Specifically, Figures 6 and 7 examined CD56^dim^NKG2A^+^ NK cells from PBMCs of HFRS patients and uninfected controls, focusing on their baseline expression levels. In contrast, Figure 11 analyzed the expression of TNF-α, perforin, and CD107a on purified NK cells following co-culture with K562/HLA-E cells pulsed with HTNV-derived peptides (NP5). This *ex vivo* co-culture assay is designed to investigate the impact of specific HTNV-derived peptides on the antiviral functions of CD56^dim^NKG2A^+^ NK cells, providing insights into the mechanisms underlying the interaction between HLA-E/peptide complexes and the CD94/NKG2A receptor.

In the revised manuscript, we have incorporated these findings into the Results section (Page 12, Line 247 to Page 15 Line 289) and presented them as a new figure (Figs 6 and 7). This integration allows for a more comprehensive comparison and clearer presentation of the data. We have also added a detailed discussion to address these inconsistencies and provide a more thorough explanation of the observed trends.

Page 12, Line 247 to Page 15 Line 289:

“**CD56^dim^NKG2A^+^ NK Cells in HFRS Patients Correlate with Disease Severity and Clinical Course**

We investigated the production of TNF-α and IFN-γ by CD56^dim^NKG2A^+^NK cells in HFRS patients. The results showed that the frequencies of TNF-α^+^CD56^dim^NKG2A^+^NK cells were significantly higher in severe/critical and mild/moderate HFRS patients compared to uninfected controls (*p*<0.05 and *p*<0.05, respectively) (Figs 6A and B). Similarly, the levels of IFN-γ produced by CD56^dim^NKG2A^+^ NK cells were significantly elevated in both severe/critical and mild/moderate HFRS patients compared to uninfected controls (*p*<0.001 and *p*<0.05, respectively) (Figs 6C and D). Furthermore, during the acute and convalescent stages, HFRS patients exhibited higher frequencies of TNF-α^+^CD56^dim^NKG2A^+^ NK cells compared to uninfected controls (*p*<0.01 and *p*<0.001, respectively) (Figs 6G and H). Notably, the production of IFN-γ by CD56^dim^NKG2A^+^ NK cells was higher in HFRS patients during the acute stage compared to those in the convalescent stage and uninfected controls (*p*<0.05 and *p*<0.001, respectively) (Figs 6I and J). The frequency of TNF-α^+^IFN-γ^+^ CD56^dim^NKG2A^+^ NK cells was also higher during the acute stage compared to the convalescent stage and uninfected controls (*p*<0.05 and *p*<0.01, respectively) (Figs 6K and L), although no significant differences were observed between different disease severity groups (Figs 6E and F).

We further examined the production of cytotoxic mediator perforin and granzyme B, as well as the degranulation of CD56^dim^NKG2A^+^ NK cells. Although no significant differences were observed between HFRS patients and uninfected controls in the frequencies of perforin^+^ or granzyme B^+^CD56^dim^NKG2A^+^ NK cells (Figs 7A-D and G-J), the expression of CD107a was significantly higher in mild/moderate and severe/critical HFRS patients compared to uninfected controls (*p*<0.01 and *p*<0.001, respectively) (Figs 7E and F). When comparing different disease stages, CD107a expression was elevated during the acute stage and remained at higher levels during the convalescent stage (*p*<0.01). The frequency of CD107a^+^CD56^dim^NKG2A^+^ NK cells was also higher at both stages compared to uninfected controls (*p*<0.05 and *p*<0.001, respectively) (Figs 7G and H).”

6. The increase of NK cell cytotoxic capacity in the presence of HTNV-derived peptide were marginal in Fig.13, cannot support the conclusion.

**Response:** Thank you very much for your insightful comments. To enhance the statistical robustness of our findings, we have expanded both the sample size (by increasing the number of acute-phase HFRS patients and uninfected controls) and the diversity of control groups (to include no-peptide, irrelevant peptide, and HLA leader peptide controls). This expansion has improved the statistical power of our comparative analyses (detailed in the revised Fig13).

Critically, our updated results confirm that CD56^dim^NKG2A^+^ NK cells specifically exhibit heightened cytotoxicity against HTNV-GP8 peptide-pulsed targets compared to HLA-Cw*03 leader peptide controls (*p* < 0.05 at an effector-to-target [E:T] ratio of 3:1). In contrast, no such enhancement was observed for CD56^dim^NKG2A^−^ NK cells or in groups stimulated with non-HTNV peptides (Figs 13B-C). Importantly, this GP8-specific activity aligns with our mechanistic findings that the four identified HTNV-derived peptides (including GP8) fail to engage the CD94/NKG2A inhibitory axis, thereby bypassing NK cell suppression and enabling degranulation. In contrast, the HLA-Cw*03 leader peptide (VMAPRTLIL), which binds to HLA-E and activates CD94/NKG2A-mediated inhibition, significantly dampened CD56^dim^NKG2A^+^ NK cell killing (*p* < 0.05 vs. GP8).

We believe these further analyses may support our conclusion that HTNV-derived peptides selectively enhance NK cell cytotoxicity by evading CD94/NKG2A-mediated inhibition. This finding is consistent with the broader understanding of NK cell biology, where the balance between activating and inhibitory signals is crucial for their cytotoxic activity.

In the revised manuscript, these data have been incorporated into a new paragraph in the Results section (Page24, Line 423 to Line 434), and Fig 13 has been added to display the results. Additionally, a thorough discussion regarding the cytolytic capacity of CD56^dim^NKG2A^+^ NK cells has been included (Page 29, Line 604 to Line 615). The experiments are described in detail in the Materials and Methods section (Page 35, Line 850 to Page 36, Line 870).

Page24, Line 423 to Line 434:

“ To evaluate the specific cytotoxic capacity of CD56^dim^NKG2A^+^ NK cells against HTNV peptide-loaded targets, we conducted comparative cytotoxicity assays between CD56^dim^NKG2A^+^ and CD56^dim^NKG2A^−^ NK cell subsets (Fig. 13A). Strikingly, at a 3:1 effector-to-target (E:T) ratio, CD56^dim^NKG2A^+^ NK cells exhibited significantly enhanced killing activity in the GP8 peptide-stimulated group compared to the HLA-Cw*03 leader peptide control (*p*<0.05), as evidenced by elevated percentages of EGFP^+^Fixable Viability Dye eFluor 450^+^ dead cells (Fig. 13B). This GP8-specific enhancement, however, was not observed in CD56^dim^NKG2A^−^ NK cells. Importantly, neither subset demonstrated differential cytotoxicity between the HLA-Cw*03 leader peptide control and groups lacking peptide stimulation, exposed to alternative control peptides, or treated with other HTNV-derived peptides (Figs. 13B and C), highlighting the selective response to GP8 peptide in CD56^dim^NKG2A^+^ NK cells.”

Page 29, Line 604 to Line 615:

“Furthermore, our findings collectively demonstrate that HLA-E-restricted presentation of the HTNV-GP8 peptide (MMKSCLIAL) selectively enhances the cytotoxic potential of CD56^dim^NKG2A^+^ NK cells by evading CD94/NKG2A-mediated inhibitory signaling. Notably, in vitro co-culture assays revealed that the HLA-E/HTNV-GP8 peptide complex significantly boosted the killing capacity of CD56^dim^NKG2A^+^ NK cells against HLA-E-expressing targets (Fig. 13), a phenomenon not observed with control peptides such as the HLA-Cw03 leader sequence (VMAPRTLIL) that actively engage the CD94/NKG2A axis. Specifically, the structural incompatibility between HTNV-derived peptides (including GP8) and the CD94/NKG2A receptor likely prevents inhibitory signaling, thereby "unshackling" CD56^dim^NKG2A^+^ NK cells to execute anti-viral cytotoxicity. These insights highlight the therapeutic potential of designing HLA-E-restricted peptides that selectively modulate NK cell responses against viral infections.”

Page 35, Line 850 to Page 36, Line 870:

“**Isolation of NK cells with FACS**

PBMCs (3×10⁷) from uninfected controls were stained with PE-Cy7-labeled anti-human CD3 mAb (Clone 17A2, BioLegend, USA), PE-labeled anti-human CD56 mAb (Clone 5.1H11, BioLegend, USA), and APC-labeled anti-human NKG2A mAb (Clone S19004C, BioLegend, USA). The cells were incubated for 30 minutes at 4°C in the dark. After incubation, the cells were washed and resuspended in RPMI-1640 medium (Gibco, USA). The CD3⁻CD56^dim^NKG2A^+^ NK cells and CD3⁻CD56^dim^NKG2A⁻ NK cells were sorted using a BD FACSAria IIU system (BD Biosciences, USA). The sorted cells were then rested in complete RPMI-1640 medium supplemented with 200 U/ml IL-2 (MedChemExpress, USA) overnight at 37°C and 5% CO₂ before the start of the killing assay.

**NK Cell Cytotoxicity Assay**

The CD3⁻CD56^dim^NKG2A^+^ NK cells and CD3⁻CD56^dim^NKG2A⁻ NK cells, sorted by FACS from PBMCs of uninfected controls, were used as effector cells. EGFP-labeled K562/HLA-E cells pulsed with HTNV-derived peptides (NP5, GP7, GP8, and GP20) were used as target cells. The effector cells were co-cultured with 1×10⁴ peptide-loaded K562/HLA-E target cells in a 96-well U-bottom plate at an effector-to-target (E:T) ratio of 3:1. Control groups included NK cells only and EGFP-labeled K562/HLA-E cells only. After a 5-hour incubation at 37°C, cells were stained for viability using Fixable Viability Dye eFluor 450 (BioLegend, USA) at the manufacturer’s recommended concentration for 30 minutes at 4°C. The cells were then analyzed by flow cytometry using an ACEA NovoExpress system (Agilent Technologies, USA). EGFP^+^ Fixable Viability Dye eFluor™ 450^+^ cells were identified as killed target cells.”

Reviewer #2: • The sample size (six HFRS patients and two controls) is relatively small. This limits the statistical power of the study and raises concerns about the generalizability of the findings. The authors should consider increasing the sample size. Otherwise, please clearly state the limitations due to the small sample size in the discussion.

**Response:** We sincerely appreciate for your critical suggestion regarding the sample size. According to the Chinese Center for Disease Control and Prevention, reported HFRS cases declined from 9,187 in 2021 to 5,218 in 2022, reflecting a nationwide reduction in disease incidence that complicated patient recruitment during our study period. Moreover, fundings constraints precluded single-cell RNA sequencing (scRNA-Seq) of additional PBMC samples, which could have provided deeper mechanistic insights.

In the revised manuscript, following the reviewer’s suggestion, we have incorporated the discussion mentioned above to provide explanations regarding the small sample size of PBMCs from HFRS patients (Page 26, Line 494 to Line 498).

Page 26, Line 494 to Line 498:

“The sample size of HFRS patients, though aligned with recent epidemiological trends in China, remains relatively small, which is an obvious limitation for this study. While our findings suggest associations between NK cell characteristics and disease severity, these observations should be interpreted as preliminary and require future validation in larger, multi-center cohorts.”

• The study demonstrates correlations between NK cell characteristics and disease severity. However, more work is needed to definitively establish causal relationships. The authors should design experiments to further validate the findings.

**Response:** We sincerely appreciate the reviewer’s comments on the need to establish more definitive causal relationships between NK cell characteristics and disease severity in HFRS. We agree that while our study provides insights into the activation and cytotoxic capacity of NK cells in HFRS, the current findings are correlative in nature. Further mechanistic studies are indeed needed to fully elucidate the underlying pathways.

To address these limitations and build on our findings, we firstly redesigned the cytotoxicity assays for NK cells to better validate the observed increase in cytotoxic capacity in the presence of HTNV-derived peptides. We expanded the sample size and introduced a more diverse set of control groups. This expansion may improve the statistical comparative analyses, as detailed in the revised Figure 13. Then we have consolidated the *ex vivo* PBMC data and *in vitro* co-culture assays into a revised Figure 7A-D for direct comparison. This integration allows for a clearer presentation of the data and facilitates a more comprehensive analysis. We also have added detailed mechanistic explanations for the peptide-specific effects observed in our study. Specifically, we identified that none of the four HTNV-derived peptides (NP5, GP7, GP8, GP20) are recognized by the NKG2A receptor, suggesting that the lack of inhibitory signaling through NKG2A leads to enhanced NK cell activity. This finding aligns with the broader understanding of NK cell biology, where the balance between activating and inhibitory signals is crucial for their cytotoxic activity.

To further validate our findings, we propose certain future direction, such as co-crystallization of HLA-E/NP5/NKG2A complexes. This structural analysis will provide a deeper understanding of the interactions between viral peptides and the NKG2A receptor. In the revised manuscript, we have addressed the limitations of our study in the Discussion section.

• While the study shows that HTNV peptides abrogate NKG2A inhibition, the precise molecular mechanisms underlying this abrogation require further investigation. The authors should explore further the interaction dynamics between HLA-E/peptide complexes and CD94/NKG2A.

**Response:** We thank the reviewer for the valuable suggestion regarding the need to further investigate the interaction dynamics between HLA-E/HTNV-derived peptide complexes and CD94/NKG2A. To address this, we plan to employ molecular dynamics (MD) simulations, which have been successfully used in several recent studies to elucidate the binding interactions and conformational dynamics of peptide-MHC complexes.

Specifically, MD simulations can provide detailed insights into how different amino acid sequences of HTNV-derived peptides affect their binding to HLA-E and subsequent interaction with the CD94/NKG2A receptor. For instance, a recent study used MD simulations to analyze the binding of various peptides to HLA-A11:01, revealing critical anchor points and the energetic profiles of these interactions. Similarly, another study utilized MD simulations to understand how specific peptide variants can interfere with the inhibitory signaling via HLA-E/NKG2A/CD94 complexes.

However, we acknowledge that our current research group lacks the experimental environment for crystallization, data collection, structure determination, and refinement necessary for such detailed structural studies. Therefore, we plan to collaborate with experts in structural biology and computational biophysics to perform these simulations and analyze the interaction dynamics in our future studies. This collaboration will allow us to gain a deeper understanding of the molecular mechanisms underlying the abrogation of NKG2A inhibition by HTNV peptides and potentially identify new therapeutic targets for HFRS. We believe that these steps will significantly enhance our understanding of the mechanisms by which HTNV peptides modulate NK cell activity and contribute to the development of targeted immunotherapies. Thank you again for your valuable suggestions.

• The study focuses primarily on one HTNV peptide (NP5). Further investigation with other identified peptides is necessary to establish whether this effect is specific to NP5 or a common property of multiple HTNV peptides.

**Response:** We sincerely thank the reviewer for raising this critical point regarding the functional characterization of the NP5 peptide. As requested, we have now incorporated comprehensive analyses of all four HTNV-derived peptides (NP5, GP7, GP8, and GP20) into the revised manuscript to clarify their distinct effects on NK cell activity.

First, NP5 exhibits high HLA-E binding affinity but fails to engage CD94/NKG2A inhibition. In our HLA-E*0103 binding assays, NP5 demonstrated the highest binding affinity among the four HTNV-derived peptides (fluorescence intensity [FI] = 2.20, Table 2). Despite this strong HLA-E interaction, NP5 did not suppress CD56^dim^NKG2A^+^ NK cell cytotoxicity in functional assays (revised Fig. 13B-C). This contrasts with the HLA-Cw*03 leader peptide (VMAPRTLIL), which robustly inhibited NK cell activity via CD94/NKG2A engagement. The dissociation between HLA-E binding and inhibitory signaling suggests that NP5 lacks structural complementarity to the CD94/NKG2A receptor, a prerequisite for initiating inhibitory signaling. Second, due to initial funding limitations, NP5 was prioritized for early experiments based on its high HLA-E binding capacity (Table 2) and its reported immunogenicity in CD8^+^ T cell responses.

In the revised manuscript, we have now expanded all functional assays to include all four peptides (NP5, GP7, GP8, GP20). These data are presented in revised Figures 12–13 and described in the Results section (Page 22, Line 399 to Page 25, Line 434). Our expanded analyses now include all four HTNV-derived peptides, providing a more comprehensive understanding of their effects on NK cell activity. We believe these revisions address the reviewer's concerns and enhance the robustness of our study. Thank you again for your valuable comments.

Page 22, Line 399 to Page 25, Line 434:

“To further the functional phenotypes and indicators across different peptide groups. Our results demonstrated that CD56^dim^NKG2A^+^NK cells from uninfected controls, when co-cultured with K562/HLA-E cells loaded with HTNV peptides, exhibited significantly higher levels of CD107a expression compared to those co-cultured with cells presenting the HLA-Cw*03 leader peptide control (*p*<0.05 and *p*<0.01, respectively) (Figs 12A and B). Moreover, we compared the degranulation activity between CD56^dim^NKG2A^+^ and CD56^dim^NKG2A^−^NK cells. In contrast, CD56^dim^NKG2A^−^ NK cells did not exhibit significant differences in degranulation across the various groups (Figs 12C and D). Additionally, the expression of perforin and TNF-α in both CD56^dim^NKG2A^+^ and CD56^dim^NKG2A^−^ NK cells remained unchanged across all groups (Figs 12E-L).

**The HLA-E/HTNV-derived peptide complex enhanced the cytotoxic capacity of CD56^dim^NKG2A^+^NK cells at the acute stage of HFRS**

To evaluate the specific cytotoxic capacity of CD56^dim^NKG2A^+^NK cells against HTNV peptide-loaded targets, we conducted comparative cytotoxicity assays between CD56^dim^NKG2A^+^ and CD56^dim^NKG2A^−^NK cell subsets (Fig 13A). Strikingly, at a 3:1 effector-to-target (E:T) ratio, CD56^dim^NKG2A^+^NK cells exhibited significantly enhanced killing activity in the GP8 peptide-stimulated group compared to the HLA-Cw*03 leader peptide control (*p*<0.05), as evidenced by elevated percentages of EGFP^+^Fixable Viability Dye eFluor™ 450^+^ dead cells (Fig 13B). This GP8-specific enhancement, however, was not observed in CD56^dim^NKG2A^−^NK cells. Importantly, neither subset demonstrated differential cytotoxicity between the HLA-Cw*03 leader peptide control and groups lacking peptide stimulation, exposed to alternative control peptides, or treated with other HTNV-derived peptides (Figs 13B and C), highlighting the selective response to GP8 peptide in CD56^dim^NKG2A^+^ NK cells.”

• The authors should expand the control groups to include healthy individuals without evidence of HTNV exposure or other relevant viral infections to better account for confounding factors.

**Response:** We fully agree with your valuable suggestions. To address this concern, we have expanded our control groups to include healthy individuals who have no evidence of HTNV exposure or other relevant viral infections. This expansion allows us to more accurately assess the specific effects of HTNV infection on NK cell activity, as opposed to general immune activation seen in other viral infections.

First, we have included additional uninfected controls who have been screened for HTNV exposure using specific serological assays. These individuals have no detectable antibodies against HTNV, ensuring that our control group is free from prior HTNV exposure. Second, to account for potential confounding effects from other viral infections, we have also included individuals with documented exposure to other relevant viruses, such as influenza and cytomegalovirus, but without HTNV exposure. This allows us to differentiate the effects of HTNV-specific infection from general immune activation seen in other viral contexts.

To further validate our findings, we have conducted additional experiments using these expanded control groups. Specifically, we have performed co-culture experiments with PBMCs from both uninfected controls and individuals with other viral infections, using peptide-pulsed K562/HLA-E cells. The results show that HTNV-derived peptides specifically enhance the cytotoxic capacity of CD56^dim^NKG2A^+^ NK cells, an effect not observed in controls without HTNV exposure. Moreover, we have expanded our cytotoxicity assays to include sorted CD56^dim^NKG2A^+^ NK cells from these additional control groups. The data confirm that the enhanced cytotoxicity observed in HTNV-infected individuals is specific to HTNV peptides and not a general feature of NK cells from individuals with other viral infections.

Reviewer #3: Figure 3 - NKG2A is higher in cells from HFRS patients. What is mediating this and could it simply be the higher amounts of NKG2A and not its engagement by peptide bearing HLA-E is driving the enhanced disease?

**Response:** Thank you very much for your insightful question. The elevated expression of NKG2A in HFRS patients is likely multifactorial and not solely due to the recognition of HLA-E presenting viral peptides. Several studies have shown that various tumor cells upregulate HLA-E expression to enhance binding to NKG2A, thereby inhibiting the cytotoxic function of NK cells and promoting tumor immune evasion (ref 1-2). Similarly, in viral infections, the NKG2A-HLA-E axis plays a crucial role in immune evasion. For instance, the overexpression of HLA-E in viral-infected cells can lead to increased NKG2A expression on NK cells, which in turn suppresses their cytotoxic activity. NKG2A contains two immunoreceptor tyrosine-based inhibition motifs (ITIMs) in its cytoplasmic region, which can recruit intracellular phosphatases SHP-1 and SHP-2. Upon binding to HLA-E, NKG2A induces an inhibitory signal that suppresses the activation of both CD8^+^ T cells and NK cells. This inhibitory signaling is critical for the immune evasion strategies employed by pathogens and tumors.

In the context of HFRS, the higher expression of NKG2A in NK cells could be driven by several factors. First, HLA-E expression is generally elevated in infected cells, including those infected with HTNV. This upregulation can lead to increased NKG2A expression as a compensatory mechanism to inhibit NK cell activity. Second, the cytokine milieu during HTNV infection might also contribute to NKG2A upregulation. For example, IFN-γ, which is often produced in response to viral infections, can upregulate HLA-E expression, thereby increasing NKG2A engagement. Third, NKG2A expression is also involved in NK cell education and maintenance of their expansion capacity. The higher NKG2A expression in HFRS patients might reflect an adaptive response aimed at maintaining NK cell functionality while avoiding excessive activation and exhaustion. Our future studies should focus on dissecting these mechanisms to better understand the role of NKG2A in HFRS pathogenesis and immune response.

Reference:

Ref 1. Liu X, Song J, Zhang H, Liu X, et al. Immune checkpoint HLA-E:CD94-NKG2A mediates evasion of circulating tumor cells from NK cell surveillance. Cancer Cell. 2023;41(2):272-287.e9.

Ref 2. André P, Denis C, Soulas C, Bourbon-Caillet C, et al. Anti-NKG2A mAb Is a Checkpoint Inhibitor that Promotes Anti-tumor Immunity by Unleashing Both T and NK Cells. Cell. 2018;175(7):1731-1743.e13.

Figure 6 legend and lines 248-250 - Since the cytokines analyzed are IFNg and TNFa , the word "cytokines" should be replaced with "IFNg and TNFa" unless the authors are willing to look for more cytokines.

**Response:** We accept these valuable suggestions. As suggested, we have replaced the word "cytokines" with "IFN-γ and TNF-α" in the manuscript (such as Page 1, Line 11 to Line 13 and Page 17, Line 380 and Page 22).

Page 1, Line 11 to Line 13:

“In this study, CD56^dim^CD16^+^NKG2A^+^ NK cells were the main subset in HFRS patients, showing activation and proliferation phenotypes with NKG2C^-^CD57^-^ and the ability to secrete tumor necrosis factor-α (TNF-α), interferon-γ (IFN-γ) and cytotoxic mediators.”

Figure 11 - Can the authors use a peptide known to induce an inhibitory effect on NK cells in this experiment to show that inhibition of NK cell activation is possible in this assay?

**Response:** Thank you very much for your genuine comment. We appreciate your suggestion regarding the use of a peptide known to induce an inhibitory effect on NK cells in our experiments. This is an important consideration to validate the assay setup and ensure that the observed effects are specific to the HTNV-derived peptides.

Previous studies have demonstrated that the peptide from the HLA-Cw03 leading sequence (VMAPRTLIL) stabilizes HLA-E complexes and induces an inhibitory effect on NK cells through specific interaction with the CD94/NKG2A receptor (ref 1). This peptide has been widely used as a control in studies examining the inhibitory effects of HLA-E on NK cell activation. In our experiments, we have used this HLA-Cw*03 leader peptide as a control to demonstrate that inhibition of NK cell activation is indeed possible in our assay. Specifically, we observed that the HLA-Cw*03 leader peptide significantly inhibited NK cell activation, confirming that our assay is capable of detecting inhibitory effects. This control peptide was included in all sections of our study, particularly in the cytotoxicity assays, to ensure the reliability and specificity of our findings.

Reference:

Ref 1. Tarragó D, González I, González-Escribano MF. HLA-E restricted cytomegalovirus UL40 peptide polymorphism may represent a risk factor following congenital infection. BMC Genomics. 2022;23(1):455.

**Part III – Minor Issues: Editorial and Data Presentation Modifications**
Please use this section for editorial suggestions as well as relatively minor modifications of existing data that would enhance clarity.

Reviewer #1: The manuscript was poorly written, with many writing errors, such as “Patents” in line 604; “CD3D, FCER1G, FCERIG, NCR1” in line 123. The figure legends need more details about how they performed the experiments.

**Response:** We are grateful to the reviewer for pointing out this error. The minor error of writing and describing in the figure legend would make the readers confusion to understand the result. We have carefully checked the writing errors (Page 30, Line 642 and Page 4, Line 123) and added more details about how we performed the experiments in the figure legends (Page 21, Line 390 to Page 22, Line 398, Line 395, Page 23, Line 411 to Page 24, Line 420 and Page 25, Line 436 to Line 446) as follows.

Page 30, Line 642:

“**Patients**”

Page 4, Line 123:

“*CD3D*, *FCER1G*, *NCAM1*, *NCR1*”

Page 21, Line 390 to Page 22, Line 398:

“**Fig 11. Activation, proliferation and effector capacities of CD56^dim^NKG2A^+^NK cells in HFRS patients by co-culture with HTNV-NP5 peptide-pulsed K562/HLA-E*0103 cells *ex vivo***.

Representative flow cytometric plots and changes in the frequencies of activated (HLA-DR^+^) (A,B), proliferated (Ki67^+^) (C,D) and functional (TNF-α^+^, perforin^+^ and CD107a^+^) (E,F) CD56^dim^NKG2A^+^NK cells between HFRS patients and uninfected controls in the presence of the HTNV-NP5 peptide. Total CD3^-^CD56^+^NK cells were isolated from the PBMCs of each HFRS patient and uninfected control using a human NK cell negative selection kit. The isolated NK cells were then co-cultured with HTNV-NP5 peptide-pulsed K562/HLA-E*0103 cells *ex vivo.* Statistical analysis was performed using the Mann-Whitney *U* test, with p-values below 0.05 considered statistically significant (*p* < 0.05, *; *p* < 0.01, **).”

Page 23, Line 411 to Page 24, Line 420:

“**Fig 12. Activation, proliferation, TNF-α and cytotoxic mediator production of CD56^dim^NKG2A^+^NK cells upon co-culture with K562/HLA-E cells pulsed with the indicated peptides *ex vivo*.**

Representative flow cytometric (FCM) analysis and cumulative results showing the frequencies of CD107a^+^ (A-B), perforin^+^ (C-D) and TNF-α^+^ (E-F) in CD56^dim^NKG2A^+^ and CD56^dim^NKG2A^-^NK cells from PBMCs of uninfected controls across different peptide groups. K562/HLA-E*0103 cells loaded with peptide from HLA-Cw*03 leading sequence (VMAPRTLIL) were used as the positive control, while K562/HLA-E*0103 cells loaded with the peptide from HCMV phosphoprotein PP65 (NLVPMVATV) or no peptide were used as negative controls. Statistical analysis was performed using the Mann-Whitney *U* test, with p-values below 0.05 considered statistically significant (*p* < 0.05, *).”

Page 25, Line 436 to Line 446:

“**Fig 13. Ability of the HLA-E/HTNV-derived peptide complex to induce CD56^dim^NKG2A^+^NK cell-mediated lysis**.

(A) Representative flow cytometric plots and (B) Kinetics and comparison showing the percentages of targeted cells killed by CD56^dim^NKG2A^+^ and CD56^dim^NKG2A^-^NK cells at effector-to-target cell ratios of 3:1 in different peptides groups. EGFP-labeled K562/HLA-E*0103 cells loaded with indicated peptide were used as target cells, while FACS-sorted CD3^-^CD56^dim^NKG2A^+^ and CD3^-^CD56^dim^NKG2A^-^NK cells from the PBMCs of uninfected controls were used as effector cells. K562/HLA-E*0103 cells loaded with the peptide from the HLA-Cw*03 leading sequence (VMAPRTLIL) served as the positive control, whereas K562/HLA-E0103 cells loaded with the peptide from HCMV phosphoprotein PP65 (NLVPMVATV) or no peptide served as negative controls. Fixable Viability Dye eFluor™ 450 was used to label dead cells.”

Reviewer #2: • Some figures are complex and could benefit from clearer labeling, improved visual presentation, and more concise legends. The authors should consider consolidating or simplifying some of the figures.

**Response:** Thank you for your valuable suggestions regarding the complexity of some figures and the need for clearer labeling and improved visual presentation. We have taken your suggestions seriously and have made several revisions to enhance the clarity and conciseness of our figures. For example, Figures 3-7 and Figures 11-13 have been revised to present the data more succinctly while retaining all essential information. We also have simplified the visual presentation of the figures by using clearer labeling.

Reviewer #3: Lines 26-29 - There is a lot speculation in this sentence.
With all six infected patients being males but one of the two uninfected patients being female, it is important for the authors to make some statements about sex-differences in HFRS and how this skewing of their study populations might impact the significance of their results.

**Response:** Thank you very much for pointing out the important limitation regarding the gender distribution in our study population. We appreciate your suggestion and have considered the epidemiological studies of HFRS, which have reported significant differences in gender distribution.

Epidemiological studies have consistently shown a higher incidence of HFRS among males compared to females. For instance, a study conducted in Zhejiang Province reported a male-to-female ratio of 2.6:1 among HFRS patients across all age groups (Ref 1). Similarly, data from Jilin Province between 2018 and 2023 revealed a gender ratio of 3.13:1, with a total of 1,511 male cases and 482 female cases reported (Ref 2). This gender disparity is likely related to occupational and behavioral patterns. Farming and manual labor, which are the primary occupations associated with HFRS infection, are more commonly performed by men. This increased exposure to rodents carrying HTNV or their bodily fluids may contribute to the higher incidence rate of HFRS among men compared to women.

Given the gender skew in our study population, with all six infected patients being male and only one of the two uninfected patients being female, we acknowledge that this may impact the generalizability of our findings. To address this, we have added a discussion on the potential implications of this gender imbalance in our revised manuscript. Specifically, we have included a statement on Page 26, Line 489 to Line 494, highlighting the importance of considering sex differences in HFRS and the potential impact of our study population's gender distribution on the significance of our results.

We have also adjusted the interpretation of our results to avoid overgeneralization. The revised results are presented on Page 6, Line 144 to Line 146, and include a note on the limitations related to the gender distribution of our study population.

To address the editor’s concern about overstating conclusions in Author Summary, we have revised the text to temper claims about the effects and mechanisms of NKG2A-HLA-E axis-mediated NK cell responses in HTNV infection disease. For example, in the original sentence" Here, the findings demonstrate that the elevated percentage of CD56^dim^NKG2A^+^ NK cell subset in peripheral blood of HFRS patients might exert antiviral effects through the unrecognize between CD94/NKG2A and HLA-E/HTNV peptide complex, which may abrogate the inhibition of NKG2A-expressing NK cells.*"* we have rephrased it to (Page 1, Line 27 to Line 30): " Here, the findings suggest a potential link between NKG2A-expressing NK cell functional profiles and HTNV infection. The link may be mediated by the mechanism of the lack of recognition between CD94/NKG2A and the HLA-E/HTNV peptide complex."

Page 6, Line 144 to Line 146:

“**Two distinct subclusters of NK cells exhibited high expression of NKG2A and displayed signatures associated with inflammation and proliferation during HTNV infection** ”

Page 26, Line 489 to Line 494:

“Epidemiological studies of HFRS have consistently reported a significant gender disparity in the incidence of the disease. For example, a study conducted in Zhejiang Province found that the male-to-female ratio among HFRS patients across all age groups was 2.6:1. Similarly, data from Jilin Province between 2018 and 2023 revealed a pronounced predominance of male patients, with a total of 1,511 male cases compared to 482 female cases, resulting in a gender ratio of 3.13:1.”

Reference:

Ref 1. Zhang R, Mao Z, Yang J, Liu S, et al. The changing epidemiology of hemorrhagic fever with renal syndrome in Southeastern China during 1963-2020: A retrospective analysis of surveillance data. PLoS Negl Trop Dis. 2021;15(8):e0009673.

Ref 2.WANG Junfeng, WU Hao, LI Meina, ZHAO Qinglong. Analysis on the epidemiological characteristics of hemorrhagic fever with renal syndrome and surveillance results of animal hosts in Jilin Province from 2018 to 2023. Journal of Tropical Diseases and Parasitology, 2024, 22(3): 133-139.
